# Protective Effects of Controlled Mechanical Loading of Bone in C57BL6/J Mice Subject to Disuse

**DOI:** 10.1002/jbm4.10322

**Published:** 2019-12-27

**Authors:** Alex DeLong, Michael A Friedman, Scott M Tucker, Andrew R Krause, Allen Kunselman, Henry J Donahue, Gregory S Lewis

**Affiliations:** ^1^ Department of Comparative Medicine Pennsylvania State University, College of Medicine Hershey PA USA; ^2^ Department of Orthopaedics & Rehabilitation, & Center for Orthopaedic Research and Translational Science Pennsylvania State University, College of Medicine Hershey PA USA; ^3^ Department of Public Health Sciences Pennsylvania State University, College of Medicine Hershey PA USA; ^4^ Department of Biomedical Engineering Virginia Commonwealth University Richmond VA USA

**Keywords:** BONE, μCT, HINDLIMB SUSPENSION, MECHANICAL LOAD, OSTEOPOROSIS, STRAIN

## Abstract

Prolonged reduction in weightbearing causes bone loss. Disuse of bone is associated with recovery from common musculoskeletal injury and trauma, bed rest resulting from various medical conditions, and spaceflight. The hindlimb‐suspension rodent model is popular for simulating unloading and disuse. We hypothesized that controlled mechanical loading of the tibia would protect against bone loss occurring from concurrent disuse. Additionally, we hypothesized that areas of high mechanical peak strains (midshaft) would provide more protection than areas of lower strain (distal shaft). Adult C57BL6/J mice were suspended for 3 weeks, with one limb subjected to tibial compression four times per week. μCT imaging was completed at days 0, 11, and 21, in addition to serum analysis. Significant bone loss caused by hindlimb suspension was detected in trabecular bone by day 11 and worsened by day 21 (*p* < 0.05). Bone loss was also detected in cortical thickness and area fraction by day 21. However, four short bouts per week of compressive loading protected the loaded limb from much of this bone loss. At day 21, we observed a 50% loss in trabecular bone volume/total volume and a 6% loss in midshaft cortical thickness in unloaded limbs, but only 15% and 2% corresponding losses in contralateral loaded limbs (*p* = 0.001 and *p* = 0.02). Many bone geometry parameters of the loaded limbs of suspended animals did not significantly differ from non‐suspended control limbs. Conversely, this protective effect of loading was not detected in cortical bone at the lower‐strained distal shaft. Analysis of bone metabolism markers suggested that the benefits of loading occurred through increased formation instead of decreased resorption. This study uniquely isolates the role of externally applied mechanical loading of the mouse tibia, in the absence of muscle stimulation, in protecting bone from concurrent disuse‐related loss, and demonstrates that limited bouts of loading may be highly effective during prolonged disuse. © 2019 The Authors. *JBMR Plus* published by Wiley Periodicals, Inc. on behalf of American Society for Bone and Mineral Research.

## Introduction

Prolonged disuse or limited weightbearing can cause significant detrimental effects on bone mass and strength. Disuse osteoporosis is a common skeletal disorder of the elderly and in patients subjected to prolonged immobility or bed rest, which can go unnoticed until it presents as fractures or spinal injuries.^1^, ^2^, ^3^, ^4^, ^5^ The weight‐bearing lower extremities are predominantly affected by osteoporosis in disuse cases caused by a lack of adequate loading.^6^, ^7^ These conditions can lead to decreased mobility, reduced daily activities, lower quality of life, and increased risk for falls and fractures. Similarly, spaceflight can induce negative effects on bone and muscle.^8^, ^9^, ^10^, ^11^ One year after returning to Earth, the femoral BMD of astronauts who had spent 4 to 6 months on the International Space Station had only partially recovered.^8^, ^10^, ^12^

Therapeutic interventions, such as pharmacological, exercise, and nutritional strategies, have yielded variable success. Several countermeasures that have theoretical potential include use of prescription drugs (eg, bisphosphonates and parathyroid hormone) and training regimens that have a significant resistance‐exercise component.^13^ Leblanc and colleagues reported that alendronate, a bisphosphonate that inhibits osteoclastic bone resorption, attenuates most of the bone loss associated with long duration of bed rest.^9^, ^14^ Furthermore, this group reports that bisphosphonates, as a supplement to exercise, can protect bone during long‐duration spaceflight.^9^ Recently, there have been contrary reports to the effect of alendronate on bone‐formation rates.^15^, ^16^ There have also been variable reports of resistance exercise to be a countermeasure to disuse‐induced bone loss.^13^, ^15^, ^16^, ^17^, ^18^, ^19^ Leblanc and colleagues utilized a newer advanced resistance‐exercise device with bisphosphonates to demonstrate the protection of bone during spaceflight with increases from BMD scans, elevated measures of bone‐resorption markers, and urinary excretion of calcium.^9^, ^20^ Shackelford and colleagues used a specially designed and NASA‐developed horizontal exercise machine as a method of resistance exercise for patients undergoing 17 weeks of horizontal bed rest.^13^ Patients using the resistance‐training protocol had significantly different lumbar, hip, calcaneus, and pelvis BMDs, indicating that the exercise regime had a positive treatment affect for patients in situations such as prolonged bed rest and spaceflight.^13^ Schneider and colleagues reported that astronauts training with an interim resistance‐exercise device demonstrated significant increases in muscle strength and volume; however, no significance was detected in BMD.^10^ Smith and colleagues evaluated the use of an “interim resistive exercise device” and nutrient analysis to demonstrate no significant differences in BMD from “advanced resistive exercise device” crewmembers.^21^ Lam and Qin utilized frequency‐dependent dynamic muscle stimulation to inhibit trabecular bone loss in a hindlimb‐suspended rat model.^1^ These studies included muscle contractions in conjunction with loading of bone, with consequential muscle‐bone “cross‐talk,” including biomechanical and secreted signaling molecules. Conversely, there are limited studies published, that utilize externally applied bone‐loading (without muscle contraction) in conjunction with a general state of disuse.^22^ This form of loading can help isolate the role of bone mechanical stimulus, such as mechanical strain in preventing bone loss, from the confounding muscle effects.

The hindlimb‐suspension unloading rodent model has been widely used for simulating unloading associated with spaceflight, and now is also used extensively to investigate muscle atrophy and disuse osteopenia caused by Earth‐based conditions such as muscle‐wasting disease, inactivity, bed rest, and immobilization.^23^, ^24^ In this model, the hindlimbs are freely moveable unlike other models of disuse, such as spinal cord injury, neurectomy botoxilin, or casting, in which the hindlimbs are functionally immobilized. Previous experiments showed that hindlimb‐suspended C57BL/6J mice had significant differences in cortical and trabecular femoral and tibial bone when compared with control animals at 21 days.^8^ It was also found that there were significant decreases in the absolute mass of gastrocnemius and quadriceps muscles, increases in atrogenes, MuRF1 and Atrogin‐1, and a significant decrease in muscle protein synthesis on days 7 to 14.^8^ There are numerous other reports in the literature reporting significant bone and muscle loss with the hindlimb‐suspension model with as little as 14‐day suspension.^8^, ^13^, ^23^, ^24^, ^25^

In vivo mechanical loading, creating higher strain environments, can induce an osteogenic atmosphere. in vivo loading models of turkey radius/ulna, rat femur/tibia, and murine femur/tibia have been reported.^12^, ^25^ The tibial‐compression loading model is a popular noninvasive method shown to induce an anabolic response in both trabecular and cortical bone compartments. This model applies cyclic loads that include both physiologically relevant axial and bending modes.^26^, ^27^

Although bone strain distributions resulting from tibial compression are complex, certain generalities can be made according to basic mechanics theory and findings from numerical modeling and experimental measurements: both traditional strain gauges and newer approaches such as digital image correlation.^28^ Under tibial compression, the mouse tibial midshaft and regions proximally are exposed to relatively high tensile and compression peak longitudinal strains. These higher strains are caused mainly by eccentric bending, which is induced by the fact that the longitudinal axis of the proximal half of the tibia is offset from the axis of compression. Conversely, the portion of the tibia distal to the tibia–fibula junction is more in line with the compression axis and not exposed to these high bending moments, resulting in lower magnitude compressive strains, with peak magnitudes on the order of half of those at the midshaft.^28^

The differential between usual and novel strains has been reported to be most relevant to mechano‐responsiveness.^29^ Regions routinely exposed to lower strains could require a lower strain to reach a cellular osteogenic threshold.^30^ However, strains during normal cage activity are more variable than those during tibial axial compression and include bending and potentially torsion.^30^

The objective of this study was to examine the role of controlled mechanical loading in preventing bone loss associated with an otherwise catabolic state of bone disuse. It was hypothesized that limited bouts of mechanical loading would be protective from bone loss induced from hindlimb suspension, and secondarily that loading in cortical areas of high peak strain (midshaft) would provide protection, but loading in areas of lower strain (distal shaft) would not. Bone parameters were determined using μCT analysis and serum biomarkers. These hypotheses were tested by using the hindlimb‐suspension model in combination with the in vivo mechanical tibial‐loading model in C57Bl/6J mice. Although the hindlimb‐suspension and tibial‐compression models have been used individually, to our knowledge they have never been utilized simultaneously.

## Subjects and Methods

### Animals

Male WT C57BL/6J mice (Jackson Laboratories, Bar Harbor, ME, USA) were used. All mice were approximately 112 days (16 weeks) ± 3 days (25 g ± 2 g) at experimental outset, and therefore defined as skeletally mature at day 0 of the experiment. Mice were allocated randomly into experimental groups with internal controls for determining the primary effect of tibial compression (Table 1). A separate group of hindlimb‐suspended‐only mice, no load or anesthesia, was used as controls for serum analysis. All mice were acclimated to the room used for hindlimb suspension according to the institutional acclimation protocol. Mice were housed in standard polycarbonate enclosures modified for hindlimb suspension (2 mice/cage) in an animal room supplied with HEPA‐filtered air at 15 air‐changes hourly at a temperature of 25°C ± 2°C, relative humidity of 55% ± 10%, and a 12:12‐hour light/dark cycle (lights on 7:00 a.m.; lights off 7:00 p.m. with no twilight). Standard rodent 2018 Tekland Global 18% protein rodent diet (Harlan Laboratories, Indianapolis, IN, USA) and water was provided *ad libitum* throughout the experiment. All mice in the facility were screened regularly by using a health‐monitoring program and were free from a wide range of pathogens. The mice were housed in a facility accredited by the Association for Assessment and Accreditation of Laboratory Animal Care International. All animal use was performed according to the standards put forth in the *Guide for the Care and Use of Laboratory Animals* (8th ed., National Academies Press, Washington, DC; 2011), and approved by the Penn State College of Medicine IACUC.

**Table 1:** Experimental Groups and Measurements

Animal group	Hindlimb side	Name	Cage condition	Tibial compression	μCT	Serum collection
1 (*n* = 10)	Left	Control − TC	Normal ambulation	No	Days 0, 11, 21	None
	Right	Control + TC	Normal ambulation	Yes (4 d/wk)	Days 0, 11, 21	
2 (*n* = 15)	Left	HLS − TC	Hindlimb suspension	No	Days 0, 11, 21	Days 11, 21
	Right	HLS + TC	Hindlimb suspension	Yes (4 d/wk)	Days 0, 11, 21	
3 (*n* = 10)	Left/right	HLS control	Hindlimb suspension	No	None	Days 11, 21

Control = normal cage activity; HLS = hindlimb suspension; TC = tibial compression.

### Hindlimb‐suspension mechanical unloading

A modified model of hindlimb suspension was utilized, originally described by Morey‐Holton and Globus,^23^, ^24^ and previously reported.^8^, ^25^ Mice were suspended under anesthesia with isoflurane (2% delivered in 100% oxygen) at the conclusion of day 0 μCT scans. The tail was prepped with an alcohol wipe, and two strips of tape (15 cm) were applied to the tail in a helical fashion from the base of the tail for three‐quarters of the length of the tail. The pieces of tape were attached to a string used to suspend the mice to a metal bar across the top of the cage. Strings were adjusted to support the mouse at approximately 30 degrees of elevation, which has been previously described as adequate elevation for consistent hindlimb unloading while avoiding unnecessary strain on the animals.^23^, ^24^ The mice were housed two mice per cage; however, the tethering system of the hindlimb‐suspension cage prevented physical contact between the animals. Mice were inspected at least twice per day; food pellets were placed at the periphery of the cage to ensure no hindlimb‐loading occurred. Water bottles were attached to cages to provide *ad libitum* access to water. All mice received urethral cleaning with alcohol wipes twice per day to remove urethral plugs, a common observation in hindlimb‐suspended male mice. All mice were suspended for the duration of the experiment (21 days). The same caging was used to house control mice.

### Mechanical loading: in vivo tibial compression loading

On day 1 of the experiment, a noninvasive mechanical compression‐loading regime began on the right tibia of each mouse. Each mouse was loaded 4 days/week during the 21‐day experiment. The loading protocol was adapted from an osteogenic axial‐loading protocol described by Melville and colleagues.^31^ A modified anesthesia induction chamber was manufactured to ensure that no loading of the hindlimbs occurred during induction. The right leg of an anesthetized (2% isoflurane delivered in 100% oxygen) mouse was positioned in dorsal recumbency and horizontally in line with a plastic cup covered with 3/16‐inch foam padding. The cup encapsulated the flexed knee, and a rigid plastic fixture held the foot at 30 degrees dorsiflexion similar to Fritton and colleagues.^26^ Each right tibia was loaded at 1200 cycles/day with 9 N compression in a sawtooth waveform at 4 Hz, including 0.1 second dwell at 2 N between cycles. This loading protocol has previously been utilized by Yang and colleagues^32^ to demonstrate osteogenic changes in mouse tibias. Although 4 Hz is double the typical amount by mice displayed with voluntary activity, that study demonstrated that 1200 cycles per session produced greater increases in cortical area than did 36 and 216 cycles. The duration of each loading session was 5 min.

### μTomography

Mice were anesthetized with 1.5% isoflurane delivered in 100% oxygen for all μCT scans. Right and left tibias were scanned using a Scanco vivaCT 40 μCT (Scanco Medical AG, Bruttsellen, Switzerland) on day 0 as a baseline, day 11, and day 21 (at the study conclusion). Each limb was scanned in one 80‐min session. All scans were performed in vivo. Settings were 55 kVP, 145 μA, 200‐ms integration time. Image reconstruction was 2048 × 2048 matrices and isotropic voxels 10.4‐μm wide. Images were Gaussian‐filtered (sigma = 1.5, support = 2), and a 27.5% threshold was used to remove soft tissue. Trabecular sections from the proximal tibia, immediately distal to the growth plate, were evaluated over a 72‐slice region consistent with previous reports.^1^, ^25^, ^33^ Cortical sections from the midshaft and distal shaft of the tibia were evaluated, each over a 22‐slice region. These locations were identified from the total length of each tibia. The midshaft location was determined at 50% of the total tibial length and the distal location was determined at 75% tibial length. Trabecular regions were manually segmented with automated contouring within regions of interest (ROIs). Cortical region outlines (periosteal and endosteal boundaries) were segmented with a semiautomated edge‐detecting sequence in Scanco evaluation software. Data were analyzed in a manner blinded to groupings. Outcome measures for standard trabecular parameters computed with Scanco software were previously identified by Bouxsein and colleagues.^33^ These included bone volume (BV), total volume (TV), bone volume percentage (Tb.BV/TV), trabecular number (Tb.N), trabecular thickness (Tb.Th), trabecular separation (Tb.Sp), connectivity density (Conn.D), structural model index (SMI), and BMD. Cortical parameters included TV, total BV, cortical bone area fraction (Ct.BV/TV), cortical thickness (Ct.Th), cortical medullary area (Ma.Ar), cortical porosity (Ct.Po), and tissue mineral density (TMD).^33^ BMD in this case refers to the mean mineral density in the entire mixed bone–soft tissue ROI (including marrow space). To the contrary, TMD provides the information about the mineral density of only the regions segmented as bone and ignores the surrounding soft tissue, marrow, etc.

### Serum collection and analysis

Blood was collected on days 11 and 21 from two of the three groups of animals: HLS animals that were exposed to loading and the HLS only groups; blood was not collected from the control group. Approximately 250‐μL blood was collected using the submental or submandibular blood collection techniques at day 11. On day 21, blood was collected terminally from the heart after humane euthanasia. Blood samples were centrifuged, and serum was collected and stored at −80°C until serum analysis. Type I collagen N‐terminal propeptide (P1NP) and C‐terminal telopeptides (CTX) were quantified with ELISA kits (Immunodiagnostic Systems, Fountain Hills, AZ, USA) according to the manufacturer's protocols and using an Epoch Microplate Spectrophotometer (BioTek Instruments, Winooski, VT, USA). Outlier results outside the 95% confidence interval were removed.

### Statistical analyses

For the μCT data, each outcome was assessed using a general linear model with correlated errors that accounts for the longitudinal nature of the repeated measurements per animal. The general linear model with correlated errors uses restricted maximum likelihood and provides estimates of the least squares means, which differ slightly from raw observed means as the model takes into account such things as unequal group sample sizes. For each outcome, the Tukey–Kramer procedure was used to account for multiple comparisons testing between groups so that the overall family‐wise error rate was 0.05; hence, *p* < 0.05 was considered significant. The outcomes reported represent the percent change from baseline for each group of animals. For serum analysis, unpaired *t* tests were used to assess differences between groups.

## Results

### Body weight

Throughout the experiment, the mean body weight loss for the hindlimb suspension (HLS) ± tibial compression (TC) group was −8% at day 21 compared with baseline; the mean body weight loss for the control group was −5% at day 21 compared with baseline (*p* = 0.17). For each time‐point, there were no significant differences between the groups.

### Trabecular bone microstructure

**Figure 1:**
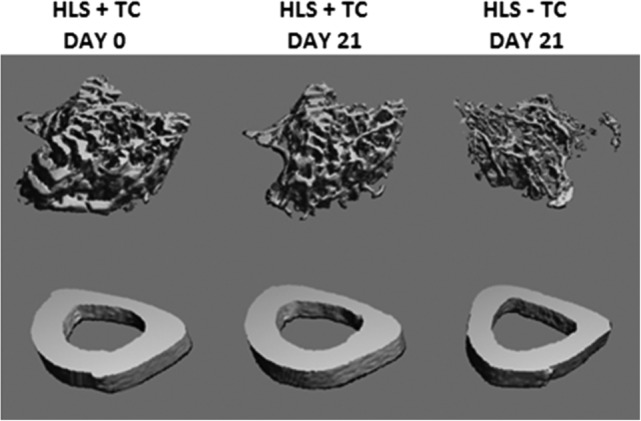
Representative 3D μCT reconstructions of trabecular (top) and cortical midshaft (bottom) microstructure. All images were obtained from the same representative animal, which was selected based on overall proximity to the mean trabecular bone volume/total volume and cortical thickness values. The animal was hindlimb‐suspended with one limb subject to tibial compression (HLS + TC) and one limb without the compression (HLS − TC). Trabecular images represent 72 slices (756 um) of proximal tibia, immediately distal to the epiphyseal plate. Cortical images represent 22 slices (231 um) of the tibia midshaft. BV/TV = bone volume/total volume; HLS = hindlimb suspension; TC = tibial compression.

**Figure 2:**
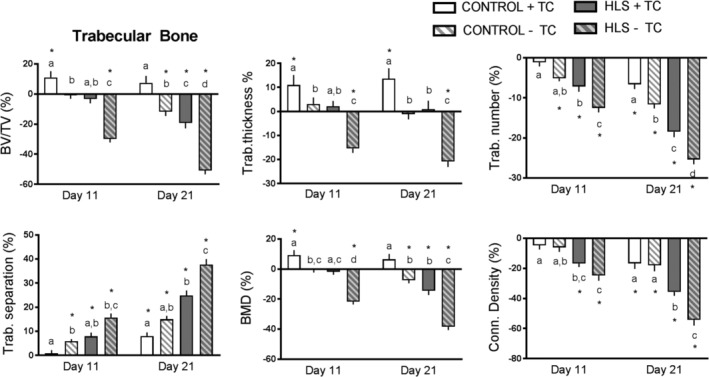
Trabecular microstructural parameters obtained from longitudinal μCT of the proximal tibia metaphysis. Data are shown as percent change from baseline (day 0), with error bars indicating ± SEM. *N* = 10 to 15/group. Significant differences between groups within time‐points are indicated by assigned letters (*p* < 0.05). Significant differences between groups and baseline are indicated by *p < 0.05.

Representative 3D images of trabecular bone are shown in Fig. 1. Hindlimb suspension (HLS − TC limb) induced a mean −30% and −51% change (relative to baseline day 0) in Tb.BV/TV at days 11 and 21, respectively (Fig. 2). These losses were significantly greater in magnitude than those associated with aging of the control mice (Control − TC limb; *p* < 0.001 at day 21), which averaged −1% and −12% at days 11 and 21, respectively. (Additional results can be found in the Supplementary Material.) Tibial compression of one limb of the hindlimb suspended animals (HLS + TC) prevented most of this disuse‐related bone loss, resulting in only −3% and −19% change in BV/TV at days 11 and 21, respectively. These losses were significantly lower at both time‐points (*p* = 0.001 at day 21) than the contralateral limb not receiving the tibial compression (HLS − TC). At day 21, the HLS + TC limb showed significantly higher loss in BV/TV (−19%) than the Control − TC limb (−12%), indicating that bone loss caused by disuse was not completely prevented by the periodic compression (*p* < 0.001). There was also a significant anabolic effect of tibial compression in the control animals (Control + TC versus Control − TC) at both time‐points (Fig. 2; *p* < 0.001 at day 21).

In general, similar results were found for other trabecular parameters (Fig. 2). Hindlimb suspension (HLS − TC) induced significantly more trabecular bone loss than the control limb (*p* < 0.05) at both time‐points and for all trabecular parameters including (in addition to BV/TV): Tb.Th (−21% at day 21 compared with baseline), Tb.N (−25%), Tb.Sp (+38%), BMD (−38%), Conn.D (−54%), and SMI (+70%). However, tibial compression of the hindlimb‐suspended animals (HLS + TC) prevented much of this loss, resulting in significant differences with the HLS − TC limb in all parameters at day 21 (*p* < 0.05), and in most parameters at day 11. For some parameters, there was no difference detected between the HLS + TC and Control − TC groups.

### Cortical midshaft bone microstructure

**Figure 3:**
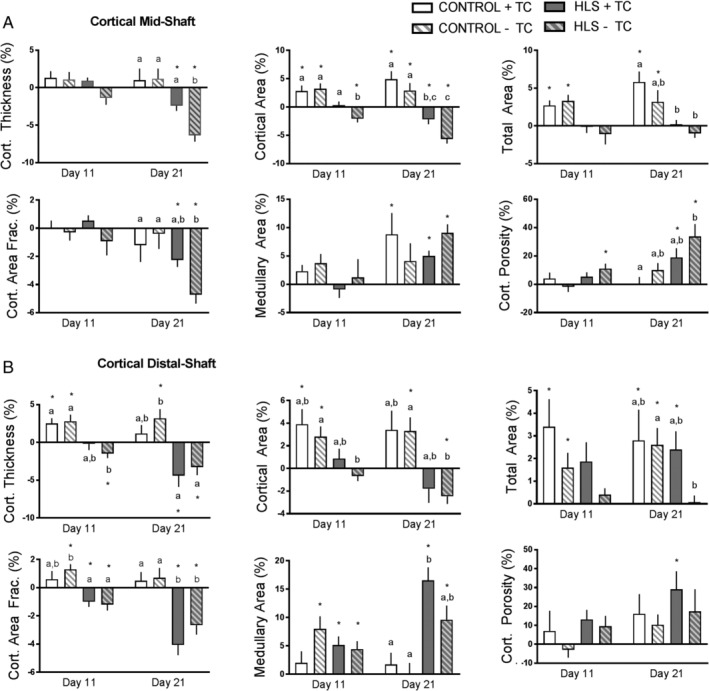
Cortical microstructural parameters obtained from longitudinal μCT of the (*A*) midshaft and (*B*) distal shaft. Data shown as percent change from baseline (day 0), with error bars indicating ± SEM. *n* = 10 to 15/group. Significant differences between groups within time‐points are indicated by assigned letters, *p* < 0.05. Significant differences between groups and baseline are indicated by *p < 0.05.

Representative images of cortical bone are shown in Fig. 1. Hindlimb suspension (HLS − TC limb) induced a mean loss in Ct.Th at the midshaft location at days 11 (−1%) and 21 (−6%), respectively (Fig. 3 *A*). The change on day 21 was significantly different than that of control mice (Control−TC limb), which had a mean gain of 1% (*p* = 0.002). Tibial compression of one limb of the hindlimb‐suspended animals (HLS + TC) prevented much of this disuse‐related bone loss, resulting in only −2% thickness at day 21, which was significantly less than the contralateral limb not receiving the tibial compression (HLS − TC; *p* = 0.024). Also, with respect to Ct.Th, at both days 11 and 21, the HLS + TC and Control − TC limbs were not significantly different, and the effects of tibial compression in the control animals were insignificant (Control + TC versus Control − TC; Fig. 3 *A*).

In general, similar trends were found for other cortical midshaft parameters (Fig. 3 *A*). For cortical midshaft μCT parameters, although significant effects of loading were observed in Ct.Th, it is notable that significant differences were not detected in cortical area, total area, or Ct.BV/TV (Fig. 3 *A*). However, there were strong trends in the cortical area and Ct.BV/TV consistent with the Ct.Th result (*p* values of 0.055 and 0.195). Total area, Ma.Ar, and Ct.Po were not significantly affected by hindlimb suspension. Although there was no significance detected with Ma.Ar, there was substantial variability in the results. Baseline porosity measurements averaged 0.76% and at the final time‐point porosity averaged 0.88% in the groups analyzed. Although there were sizeable percent changes in porosity, the porosity values themselves were less than 1%.

### Cortical distal‐shaft bone microstructure

Hindlimb suspension (HLS−TC limb) induced a mean −1% and −3% loss in Ct.Th at the distal‐shaft location at days 11 and 21, respectively (Fig. 3 *B*). This loss was significantly different than the change in control mice (Control − TC limb; *p* = 0.004 at day 21). Tibial compression of the loaded limb of the hindlimb‐suspended animals (HLS + TC) did not show significant differences compared with the unloaded contralateral limb (HLS − TC). There were also no significant effects of the tibial compression on thickness in the control animals (Control + TC versus Control – TC; Fig. 3 *B*).

In general, similar trends were found for other cortical distal‐shaft parameters (Fig. 3 *B*). Hindlimb suspension (HLS − TC) induced cortical bone loss with significant differences compared with the control limb (*p* < 0.05) at 21 days for the following parameters (in addition to thickness): cortical area (−3% at day 21 compared with baseline), total area (0%), and Ct.BV/TV (−3%). Ma.Ar and Ct.Po did not show significant effects of hindlimb suspension. At the distal location, tibial compression of the hindlimb‐suspended animals (HLS + TC) did not significantly affect any bone parameters compared with the unloaded limb (HLS − TC), and strong trends were not observed, except for total area (Fig. 3 *B*).

### Serum analysis

**Figure 4:**
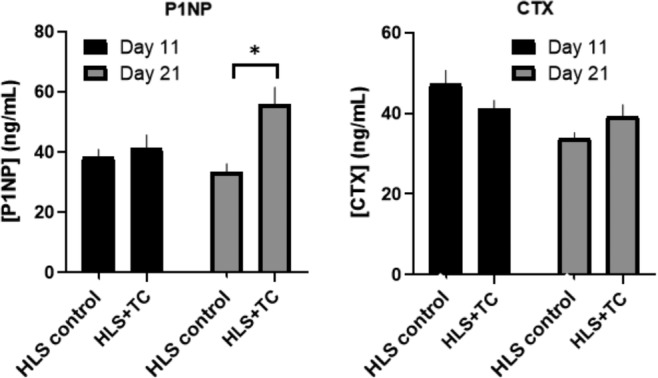
Effect of load on bone‐formation marker (P1NP) and bone‐resorption marker (CTX) in hindlimb‐suspended animals. Here HLS + TC indicates the animals that had one limb exposed to tibial compression (and one limb not), and HLS control indicates the separate group of animals that were hindlimb‐suspended without tibial compression applied to either limb. Data indicate mean, with error bars indicating ± SEM. *N* = 10 to 15/group. Significant differences between groups and time‐points are indicated by *p < 0.05.

At day 21, there was a significant increase in serum P1NP markers in the HLS + TC group (57.5 ng/mL) when compared with the separate group of HLS control animals (33.5 ng/mL; *p* = 0.009; Fig. 4). There were no significant group differences detected at day 11. There were no significant group differences detected at either time‐point for serum CTX markers.

## Discussion

The objective of the current experiment was to investigate the effects of controlled mechanical loading environments on bone loss induced by hindlimb suspension in mice. The results suggest loading of the hindlimb has a protective effect on bone loss during a chronic state of unloading. The observed effect was more evident for trabecular bone at the time‐points analyzed; however, there is evidence to support that cortical bone experiences similar, yet smaller, protective effects in the midshaft region. This is the first experiment to our knowledge to combine hindlimb suspension with a concurrent axial‐loading regime.

Bed rest, disuse, and spaceflight have historically been demonstrated to have a negative effect on bone and muscle structure and volume. Substantial losses in bone and muscle have been reported with as much as 0.5% to 1.5% bone loss per month, and as much as 10% decrease in the strength of extensor muscles of the leg.^8^, ^12^ Because of the concomitant decrease in both bone structure and muscle function, patients who experience osteopenia and sarcopenia are predisposed to catastrophic injury following such losses; therefore, effective countermeasures are needed. Bone is able to react to changing mechanical demands by adapting its internal structure through adjusting bone formation and bone resorption.^2^, ^34^, ^35^ These ideas are congruent with Wolff's Law, which states that healthy bones model and remodel regionally based on the forces that are placed upon them.

Of primary importance in our study was the paired comparison between HLS − TC and HLS + TC. The Control + TC group was anticipated to have the greatest bone quantity through the osteogenic response of loading. The Control − TC group represented age‐related bone loss as this limb would have normal weight distribution throughout the study, albeit potentially having small systemic effects from periodic anesthesia and loading the contralateral limb. The HLS − TC group, consistent with previous studies, showed significant bone loss from suspension.^8^, ^25^, ^36^

Trabecular bone was observed to be much more affected by suspension and loading than cortical bone, likely because of trabecular bone's increased turnover rate.^8^, ^25^ Interestingly, some trabecular bone parameters were not significantly different from Control − TC, including BV, SMI, Tb.Th, Tb.Sp, and BMD.^33^ These data show that the bone loss rate is not significantly different than that of a normally ambulated limb over the period of 21 days as part of the aging process. Some changes were significantly different at day 11. When comparing HLS loaded versus unloaded, BV, BV/TV, SMI, Tb.N, Tb.Th, and BMD showed significant differences. In all parameters, day 11 data show smaller absolute changes than those at day 21. This indicates that the bone loss induced from hindlimb suspension begins early and accumulates through day 21. This is consistent with previous experiments from our lab involving hindlimb suspension.^8^ Trabecular bone has long been known to show a higher response to changes in the loading environment than cortical bone. This is a suggested effect of trabeculae having the ability to optimize load transfer because of the freedom to rearrange accordingly, and because of the more rapid remodeling rate of trabecular bone.^32^

Although the number of significant cortical bone changes was less than that of trabecular bone, there was generally a protective effect at the midshaft of tibial compression in the hindlimb‐suspended animals. Data from the distal location for cortical bone, conversely, showed no significant protective effects. At the midshaft location at day 21, the most important significant difference observed was Ct.Th. The HLS − TC group showed −6% bone loss compared with −2% bone loss of the internal control or loaded limb. The other parameters showed similar trends; however, those were not significant. In all cortical parameters (mid‐ and distal shaft), there were few significant differences detected between Control − TC and HLS + TC (Ct.BV/TV [both locations] and Ma.Ar [distal location]). There were no observed significant differences in any cortical parameters at day 11, and similar to trabecular bone, the mean changes in these samples were smaller at day 11 than at day 21.

Previous experiments in C57Bl/6 mice that examined strain at midshaft showed that there was a general linear relationship between load and microstrain using load ranges of 3.8 to 11.6 N.^37^, ^38^ Previous work by De Souza and colleagues has shown little increase in bone formation when the applied compressive load is below 8 N.^27^ Although the 9 N used in this loading protocol is incrementally lower than other reported loading experiments utilizing similar protocols, the experimental subjects in this study are predisposed to a catabolic state of unloading and thus a smaller load was chosen to mitigate the risk of unnecessary injury to bone weakened by HLS.

At the midshaft location comparing the HLS groups, the Ct.Th increased with load, while the Ma.Ar also increased. This is consistent with a previous study^39^ that reported a greater marrow area in suspended mice that also received Botox injections. This result suggests that the bone formation at this location is on the periosteal surface rather than the endosteal surface.^25^ Additionally, there appears to be a delayed cortical bone response to loading and unloading, compared with trabecular bone. There is a slower turnover of cortical bone that may suggest a need for a longer experiment (ie, 4 to 6 weeks) to elucidate the cortical parameters that change during unloading and loading.^25^ Furthermore, a redistribution of calcium during remodeling from trabecular bone changes may contribute to this delayed cortical remodeling.

We surmise that high peak strain at the trabecular and midshaft cortical locations in our experiment is creating the osteogenic effect. Schulte and colleagues demonstrated that local mechanical stimuli regulate bone formation and resorption at the tissue level in mice and that bone formation most likely occurs at sites of high local mechanical strain and resorption at sites of low mechanical strain.^40^ Furthermore, a study from Sztefek and colleagues investigates bone surface strains during loading situations such as those used in this experiment. This group reports that initially in unadapted states, there are isolated areas of high strain, particularly on the medial side, and once adapted, the strains become more unified across the tibial surface.^38^ The report by Midura and colleagues demonstrates that low‐amplitude, high‐frequency strains of as little as approximately 200 με are associated with significantly higher BMD in the tibias of hindlimb‐suspended rats.^41^

Previous studies have reported that multiple methods of loading, exercise, and training are beneficial to protect bone from loss in the face of disuse. Experiments by Yanagihara and colleagues and Ju and colleagues demonstrated that continuous training in hindlimb‐suspended rats—in these cases, jumping—during a period of disuse is necessary to maintain bone quality.^42^, ^43^ Yanagihara and colleagues reported similar significant differences in tibia BMD between groups (*p* < 0.0001) to our experiment with the suspension‐only group showing 16.95% lower BMD than the training‐during‐suspension group.^42^ We demonstrated an approximate 20% difference between our groups of animals. Although examining the femoral head, Ju and colleagues also reported similar significant differences to our experiment. This group reported a decrease of 32% in Tb.N of the suspended group and an increase of 14% in Tb.Th in the jump‐exercise group, whereas we found an approximate 24% decrease in Tb.N with an increase of 20% Tb.Th, respectively.^43^ Another group, Falcai and colleagues, concluded that swimming activity not only ameliorates, but also fully prevents deleterious effects on bone quality in osteopenic rats.^44^ This group found that swimming during suspension resulted in significant increases in BMD (+43%; *p* < 0.001) compared with the 20% increase that we report. They also report increases in Tb.Th (+58%), BV/TV (+85%), and Tb.N (+27%), compared with +20% Tb.Th, +32% BV/TV, and +10% Tb.N that we report with our experiment.^44^ Falcai and colleagues also reported a lesser effect of suspension and swimming on cortical bone, similar to our findings.^44^ Swift and colleagues found similar results to the above: Trabecular bone is more affected than cortical bone. They report a 14% loss in BMD of cancellous bone in the proximal tibia from hindlimb suspension and an increase of BMD (+12%) for animals subjected to resistance training.^16^ Similar results of concurrent exercise and in this case, vibration as well, prove to be osteogenic during a state of disuse as reported by Li and colleagues.^45^

Serum biomarkers of bone formation and bone resorption may provide insight on the mechanism underlying observed tissue‐level changes. CTX is a common resorption marker of bone type I collagen by osteoclasts. Bone remodeling is a constant process that leads to an increase in circulation of type I collagen fragments associated with resorption. In a state of unloading or disuse, bone is in a constant state of resorption.^4^, ^46^ P1NP is a common bone‐formation marker formed by osteoblasts during bone remodeling. N‐ and C‐terminal extensions are removed by proteases during transformation of procollagen to collagen, creating a systemic biomarker of bone formation.^3^, ^7^, ^47^, ^48^ Together, CTX and P1NP can provide insight into the mechanism of action in a state of unloading, loading, or combination of both.^36^ Previous experiments in humans and hindlimb‐suspended rats have reported increases in P1NP with exercise; therefore, the significant increase in P1NP at day 21 in the loaded animals demonstrates that there is an anabolic effect on bone metabolism as opposed to preventing the normal catabolic cycle.^49^, ^50^ Similarly, an experiment by Bemben and colleagues demonstrated a significant increase in bone alkaline phosphatase, which represents a bone‐formation marker, a significant decrease in CTX, and an increase in BMD with high‐intensity resistance exercise.^51^ The serum biomarkers are systemic measures, whereas both the hindlimb unloading and tibia compression are site‐specific.

Melville and colleagues have detailed the in vivo tibial axial loading model.^31^ Considerations include mouse strain—WT or genetically modified—the appropriate sex based on research questions, age, loading protocol, and testing system. The amount of trabecular and cortical bone, mass, BMD, and strength can vary from one mouse strain to the next.^52^, ^53^, ^54^, ^55^, ^56^ Consideration should be made to include male, female, or both mice sexes. Aged mice are typically in a state of bone loss.^13^ It has been reported that using mice at the age of 16 weeks for loading studies may be ideal because the skeletal system is still young enough to elicit a robust anabolic response to loading, whereas at the same time, the appositional growth on the periosteal surfaces has dropped to very low levels.^31^ The effect of aging during the experiment was considered by inclusion of the control mice, and we utilized the recommended 16‐week‐old mice.

Some important limitations exist in this study. Limitations include lack of measures of local/regional bone formation/resorption, eg, dynamic histomorphometry. Longitudinal in vivo μCT is limited to providing net 3D bone formation/resorption in each animal. For the μCT, a realignment of images to account for differences in limb alignment was not performed. The study tested a single tibial compression protocol, which may not be representative of other protocols. Strain levels were not experimentally measured in this study. Despite potential crosstalk between muscle and bone, this study was limited by focusing on only bone and not muscle, such as the tibialis anterior or gastrocnemius muscle. As a potential countermeasure to disuse, the loading regime focused on bone and may not have impacted muscle mass and strength loss substantially. The serum biomarkers are limited in that they are systemic, not local, in nature and could not be compared between loaded and contralateral control limbs.

In conclusion, this study uniquely isolates the role of externally applied mechanical loading of mouse tibia, in the absence of muscle stimulation, in protecting bone from concurrent disuse‐related loss. Additionally, the study has fundamental implications for understanding the role of strain in the prevention of bone loss, especially in disuse scenarios.

## Disclosures

The authors declare that they have no conflicts of interest or disclosures in relation to the contents of this article.

## Supporting information


**Fig. S1.** Additional trabecular and cortical microstructural parameters obtained from μCT. Data shown as percent changes from baseline (day 0), with error bars indicating ± SEM. *n* = 10 to 15/group. Significant differences between groups within time‐points are indicated by assigned letters, *p* < 0.05. Significant differences between groups and baseline are indicated by *p < 0.05.

**Table S1.** Supplementary Tables.
