# Supplementary figures and images for: Protective Effects of Controlled Mechanical Loading of Bone in C57BL6/J Mice Subject to Disuse
Source: JBMR Plus. 2019 Dec 27;4(3):e10322. doi: 10.1002/jbm4.10322 (PMC7059829; doi:10.1002/jbm4.10322)

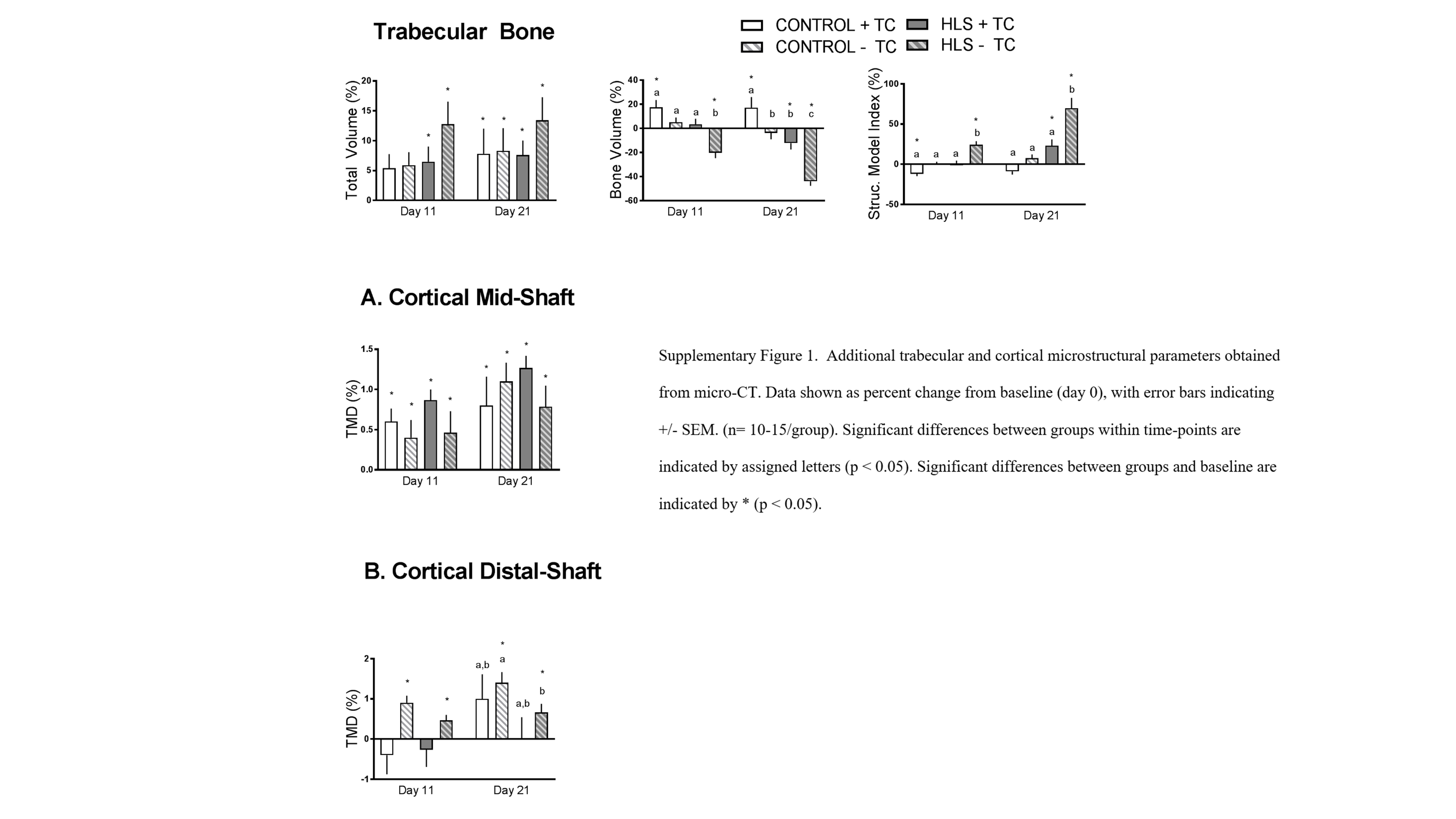

Supplement: Supplementary file 1 — Fig. S1. Additional trabecular and cortical microstructural parameters obtained from μCT. Data shown as percent changes from baseline (day 0), with error bars indicating ± SEM. n = 10 to 15/group. Significant differences between groups within time‐points are indicated by assigned letters, p < 0.05. Significant differences between groups and baseline are indicated by *p < 0.05. [file JBM4-4-e10322-s001.tif]
